# Secondary prevention after ischaemic stroke: the ASPIRE-S study

**DOI:** 10.1186/s12883-015-0466-2

**Published:** 2015-10-23

**Authors:** Linda Brewer, Lisa Mellon, Patricia Hall, Eamon Dolan, Frances Horgan, Emer Shelley, Anne Hickey, David Williams, on behalf of the ASPIRE-S Study Group

**Affiliations:** Department of Geriatric & Stroke Medicine, Royal College of Surgeons in Ireland (RCSI), Beaumont Hospital, Dublin, 9 Ireland; Department of Psychology, RCSI, Dublin, 2 Ireland; Department of Geriatric & Stroke Medicine, Connolly Hospital, Blanchardstown, Dublin, 15 Ireland; School of Physiotherapy, RCSI, Dublin, 2 Ireland; Department of Epidemiology and Public Health, RCSI, Dublin, 2 Ireland

**Keywords:** Ischaemic stroke, Secondary prevention, Risk factors

## Abstract

**Background:**

Survivors of ischaemic stroke (IS) are at high-risk for future vascular events. Comprehensive information on the adequacy of secondary prevention after IS is lacking despite the knowledge that appropriate secondary prevention improves long-term patient outcomes. ASPIRE-S (Action on Secondary Prevention Interventions and Rehabilitation in Stroke) aimed to prospectively assess secondary prevention in patients 6 months following IS.

**Methods:**

Consenting patients admitted with IS to three Dublin hospitals were recruited over 1 year, from October 2011. At 6 months post IS a comprehensive assessment was completed, modelled on the EUROASPIRE protocol for evaluation of the adequacy of secondary prevention in post-discharge cardiac patients. This assessment included measurements of blood pressure, body mass index and fasting lipid and glucose profiles. Secondary preventive medications and smoking status were also documented.

**Results:**

Three hundred two patients (58 % male) participated, of whom 256 (85 %) were followed-up at 6 months. Mean age was 69 years (range 22–95). At follow-up, 68 % of patients had a BMI >25 kg/m^2^ and 16.4 % were still smoking. Almost two-thirds (63.4 %) had a blood pressure >140/90 and 23 % had low-density-lipoprotein >2.5 mmol/L. 28 % of diabetic patients had HbA1c ≥7 %. Ninety seven percent of patients were on anti-platelet and/or anticoagulant therapy. Of those with atrial fibrillation, 82 % were anti-coagulated (mean INR of 2.4). Ninety-five percent were on lipid-lowering therapy and three-quarters were on anti-hypertensive therapy.

**Conclusion:**

This prospective multi-centre survey of IS patients demonstrated a high prevalence of remaining modifiable risk factors at 6 months post stroke, despite the widespread prescription of secondary preventive medications. There is scope to improve preventive measures after IS (in particular blood pressure) by incorporating evidence-based guidelines into quality assurance cycles in stroke care.

## Background

Stroke is a leading cause of death and disability resulting in substantial personal and healthcare costs. Although age-standardised rates of stroke mortality have decreased, the absolute number of people suffering stroke annually, stroke survivors and overall stroke burden are increasing [1]. Approximately 30 % of strokes occur in individuals with a previous stroke and 50 % occur in those with previous vascular events of any kind [2]. High recurrence rates emphasize the importance of effective preventive strategies and many population-based studies have reported strong associations between numerous cardiovascular risk factors and future stroke risk [2–4]. These findings have informed the development of evidence-based guidelines on stroke prevention [5, 6] which aim to improve stroke outcomes.

The EUROASPIRE surveys [7–9] drew attention to the continuing gap between standards set in guidelines on secondary cardiovascular disease prevention (in patients with coronary heart disease) and results achieved in clinical practice. More recently, a stroke-specific module retrospectively added to EUROASPIRE III concluded that risk factor control after IS also requires improvement [10].

We report a prospective, multi-centre study which assessed the extent to which evidence-based care was provided to a sample of Irish patients admitted with IS, as part of the Action on Secondary Prevention Interventions and Rehabilitation in Stroke (ASPIRE-S) study. This study reviewed three components of IS care following discharge, including the adequacy of secondary prevention, delivery of rehabilitative care and assessment of ongoing rehabilitative needs. This analysis focuses on the adequacy of secondary prevention at 6 months in the ASPIRE-S cohort.

## Methods

### Study hospitals and patient recruitment

Patients aged 18 years and over with IS admitted to three study hospitals were recruited between October 2011 and September 2012. Ethical approval was granted from the Medical Research Ethics Committees of participating hospitals, Beaumont Hospital (BH), Mater Misericordiae University Hospital (MMUH) and Connolly Hospital (CHB). All three study hospitals are university-affiliated teaching hospitals located in suburban (BH, CHB) and city (MMUH) North Dublin locations, serving a combined catchment area of over 750,000 people. The stroke population in North Dublin has previously been characterised by the North Dublin Population Stroke Study group (NDPSS) [11, 12], which reported that over 90 % of acute stroke cases are treated in an acute hospital. For the ASPIRE-S study, hospital-based case ascertainment was performed and a representative sample of patients with IS was sought through review of daily admissions via emergency departments and regular review of stroke consult lists in each hospital. In advance of the commencement of this study, the ASPIRE-S study team met with the stroke teams at each site to explain the study inclusion and exclusion criteria. Patients were considered eligible if they had a World Health Organisation (WHO) defined IS to include ICD-10 code 163 (cerebral infarction), including subcategories and were medically well enough for participation.

### Data collection at baseline

At recruitment, baseline details (including demographic details, stroke subtypes using TOAST [13] and Bamford [14] classifications and stroke risk factors) were collected during a short structured bedside interview. For patients who presented with severe stroke, their progress was monitored over time and their participation was requested once they were considered medically stable. Every effort was made to include patients with severe strokes, to avoid selection bias in favour of milder strokes. All eligible patients were either approached in person or (if already discharged) were phoned by a member of the research team to discuss the study and to obtain consent. Where capacity was inadequate, consent to participate was obtained from the next of kin.

### Patient interview and examination at six months

At 6 months post-stroke, patients were contacted for follow-up at which point a detailed interview and examination were carried out, using standardised methods and instruments. Most patients were reviewed in their own home by a trained member of the research team, with a minority of patients returning to a Clinical Research Centre for assessment. All patients were requested to fast from 8 pm on the preceding night for the measurement of fasting blood tests at the visit. For the purpose of this study, blood results were compared with targets outlined in the European Guidelines on cardiovascular disease prevention in clinical practice (version 2012) [15] and (for diabetes) the American Diabetes Association 2013 Guidelines [16].

### Anthropometrical and physiological measurements

#### Blood pressure

Following the measurement of arm circumference and the application of an appropriately sized cuff, patients were asked to sit comfortably with their arm relaxed and at heart level. Blood pressure (BP) was measured using a digital OMRON M6 (Intellisense™) Dual Check System. To allow for comparison with EUROASPIRE [7], the first BP measurement was recorded from the right arm, where possible. To assess for an important difference in BP between arms, we also recorded BP from the left arm. Twenty-four hour control of BP was assessed by applying a SpaceLabs 90207 monitor (SpaceLabs Inc, Wokingham, Berkshire, UK) to the arm with the highest BP reading (where possible). This measured BP every 30 min by day (8 am to 10 pm) and every 60 min overnight (10 pm to 8 am).

#### Waist circumference/BMI

Waist circumference was measured by applying a standard flexible (Farla Medical, UK) measuring tape circumferentially at the level of iliac crest and was compared with WHO-defined targets (male target <94 cm; female target <80 cm). Body weight was measured to the nearest kilogram using a calibrated (EKS International, France) mechanical weighing scales. Height was measured as the maximum distance from the floor to the vertex of the head and was recorded to the nearest centimetre. Body mass index (BMI) was calculated using the formulation weight (kg)/(height(m))^2^ and scores were categorised according to Centres for Disease Control and Prevention criteria [17].

#### Fasting blood tests

Patients had venous blood drawn to measure fasting glucose (FG) and full lipid profiles including total cholesterol (TC), low density lipoprotein (LDL), high density lipoprotein (HDL) and triglycerides (TG). Glycosylated haemoglobin (HbA1c) was measured in diabetic patients. All blood samples were processed according to local protocols governed by the Clinical Directorate of Laboratory Medicine. For patients on warfarin, recent international normalised ratio (INR) records (using patient-held booklets) were reviewed to assess the adequacy of anti-coagulation.

#### Secondary preventive medications

A full list of medications was recorded by reviewing the patient’s most recent prescription, pillbox or medication containers.

### Study governance and quality assurance

Study recruitment, follow-up and data management took place under the supervision of the study steering committee. To ensure uniformity of method the first 10 6-month visits were conducted in pairs (two of three trained researchers) and thereafter all three researchers conducted the follow-up assessments in the same manner, using identical equipment. At regular intervals throughout the study period, these assessments were conducted in pairs to assure ongoing quality and uniformity of data collection.

### Dataset and analysis

Descriptive statistics summarised and described the main findings, including percentages for categorical variables, and means and standard deviations (SD) for quantitative data. Comparisons between subgroups were performed using chi-squared analysis. Significance was calculated at a level of *p* < 0.05. Data were analysed using STATA (StataCorp 2013, Texas).

## Results

### Baseline characteristics

Three hundred two patients (mean age 69.1 years, standard deviation 12.8, range 21.8-94.9) with acute IS agreed to participate. Demographic characteristics, stroke subtype and risk factors are outlined in Table 1. Overall, patients had a mean number of three risk factors (SD 1.7; range 0-8), with almost all patients (240; 94 %) having at least one risk factor for stroke at baseline.

**Table 1:** Baseline demographic characteristics, stroke subtypes and risk factors (*N* = 302)

Demographic details	N (%)	Stroke subtype	N (%)	Risk factor	N (%)
Age		Bamford		Hypertension	176 (57)
<65	103 (34)	PACS	117 (38.7)	Hypercholesterolemia	135 (46.9)
≥65	199 (66)	LACS	81 (26.8)	Atrial fibrillation	120 (38.3)
		POCS	80 (26.5)	Heart disease	91 (29.3)
Gender		TACS	16 (5.3)	Current smoking	84 (28.1)
Male	173 (57.3)	Unclassifiable	8 (2.7)	Previous TIA/stroke	76 (25)
Female	129 (42.7)			Diabetes	60 (19.9)
		TOAST		Past smoking	53 (17.6)
Function (mRS)		Cardioembolism	121 (40.1)	Carotid disease	50 (16)
≤2	156 (51.7)	Undetermined aetiology	84 (27.8)	Alcohol excess	44 (14.5)
>3	146 (48.3)	Large vessel atherosclerosis	51 (16.9)	Depression	24 (8.2)
		Small vessel disease	33 (10.9)	Anxiety	20 (6.6)
		Other determined aetiology	13 (4.3)		

*mRS* modified rankin scale, *PACS* partial anterior circulation stroke, *POCS* posterior circulation, *LACS* lacunar stroke, *TACS* total anterior circulation stroke

Of 302 recruited patients, 46 patients (15 %) were unavailable for assessment at 6 months. Reasons for non-participation included patient refusal (*n* = 22), death (*n* = 9), intercurrent serious illness (*n* = 6), uncontactable (*n* = 5) and final diagnosis not stroke (*n* = 4). The remaining 256 patients participated in the follow-up assessment. There were no documented cases of recurrent stroke in the 6 month follow-up period for the 256 patients included.

### Risk factors at six months

#### Fasting blood results

**Fig. 1:**
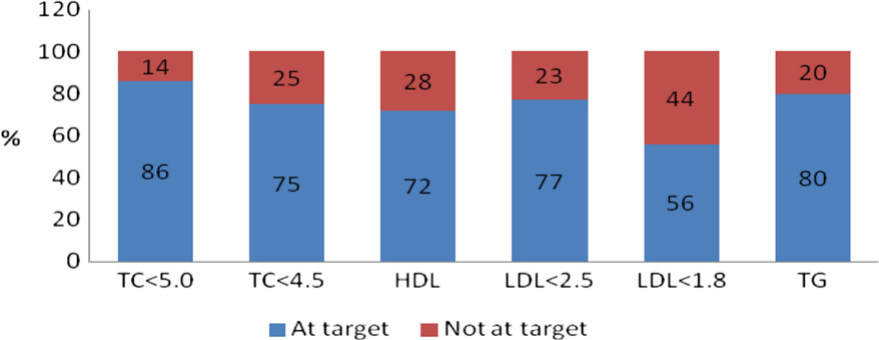
Proportions of patients with lipid results at target at 6 months; TC = total cholesterol (targets <5.0 mmol/L or <4.5 mmol/L (high-risk target) [15]); HDL = high-density lipoprotein (target >1 in males and >1.2 mmol/L in females); LDL = low-density lipoprotein (targets <2.5 mmol/L or <1.8 mmol/L (high-risk target) [15]); TG = triglycerides (target <1.7 mmol/L)

Blood was drawn from 232 patients (90.5 % of the cohort) to measure (FG) and lipid profiles. Blood was not drawn due to patient refusal, unsuccessful phlebotomy or failure to fast appropriately. Mean glucose level was 5.4 mmol/l (SD,1.35). HbA1c was measured in 91 % (*n* = 46) of diabetic patients and the mean level was 6.7 % (range 5.4–10.5; SD 0.99). Over one-quarter (28 %) of diabetic patients had an HbA1c ≥7 % and 29.4 % had FG ≥7 mmol/l. Amongst non-diabetic patients, 1.5 % had a glucose level ≥7.0 mmol/L (diabetic range), 3.9 % had a result between 6.1 and 6.9 mmol/L (impaired fasting glucose range – Europe) and 9.6 % had a result between 5.6 and 6.9 mmol/L (impaired fasting glucose range - American Diabetes Association 2013) [18]. Mean lipid values (with SD) were TC 4 mmol/l (0.99), LDL 2.1 mmol/l (0.88), HDL 1.3 mmol/l (0.37) and TG 1.4 mmol/l (0.72). The proportion of patients with fasting lipid results at target is illustrated in Fig. 1.

#### Blood pressure

##### Office blood pressure

The mean right arm systolic BP was 148 mmHg (range 92–207; SD 22.2) and the mean right arm diastolic BP was 81 mmHg (range 40–119; SD 12.8). Most patients did not reach recommended international targets for BP control (Table 2). Patients with a diagnosis of hypertension at recruitment were less likely to have their BP controlled at 6 months for each of the targets outlined.

**Table 2:** Proportion of patients with BP at target (total *N* = 249)

	Proportion of patients at target			
BP target	All patients	With diagnosed hypertension	No hypertension diagnosed	*P*-value
≤130/80^a^	40 (16.1 %)	15 (10.5 %)	25 (23.6 %)	*p* = 0.005
≤140/90^b^	91 (36.6 %)	43 (30.1 %)	48 (45.3 %)	*p* < 0.05
≤135/85^c^	69 (27.7 %)	31 (21.7 %)	38 (35.6 %)	*p* < 0.05

*BP* blood pressure

^a^Target from European guidelines on cardiovascular disease protection (high-risk patients) [15] & UK RCP National Clinical Guideline on Stroke [5] & ESH Guideline 2007 [20] (after stroke)

^b^Target used in EUROASPIRE studies [9]

^c^Target in IHF guideline on stroke [6]; *p*-values apply to comparisons between those with and without diagnosis of hypertension

##### Blood pressure in right versus left arms

A reading was unavailable from both arms in 18 (7 %) patients. Of patients with available readings bilaterally, 27 patients (11.3 %) had a discrepancy of at least 20 mmHg between systolic BP readings, an indicator of high risk for future cardiovascular events [19].

##### Ambulatory blood pressure monitoring

A 24 h ambulatory BP monitor was applied to 210 patients (82 % of participants). Thirteen patients did not wear their monitor overnight, likely due to device intolerance. Mean 24 h BP was 127/73 mmHg (systolic SD 15.6 & range 90–222; diastolic SD 9.6 & range 53–116). Daytime mean was 128/74 (systolic SD 15.7 & range 91–222; diastolic SD 10 & range 53–116) and nightime mean was 121/68 (systolic SD 15.1 & range 88–176; diastolic SD 9.7 & range 48–95). When compared with ESH Guidelines [20], more patients had their BP controlled by day (target 135/85; 61.4 %) than by night (target 120/70; 43.6 %). Fifty-nine percent had overall 24 h BP control at target (130/80).

#### Waist circumference and BMI

The mean waist circumference was 90 cm for females (*n* = 95) and 97 cm for males (*n* = 142). Two-thirds (67 %) had a waist circumference above target, including 79 % of females and 58 % of males. The mean BMI in 250 patients was 26.8 (range 17–48; SD 4.5). The proportions of patients classified as ‘normal weight (18.5–25 kg/m^2^)’, ‘overweight (25 – 30 kg/m^2^)’ and ‘obese >30 kg/m^2^)’ were 32, 44 and 24 % respectively.

#### Smoking

The proportion of patients (M:F, 60:40) that smoked 6 months post stroke was 16.4 % (*n* = 42), compared with 28 % (*n* = 72) at baseline.

### Secondary preventive therapy

#### Antithrombotic medications

Almost all (249; 97.3 %) were on an anti-platelet and/or anticoagulant medication. 160 patients (62.5 %) were on anti-platelet medication and, of these, 36 patients (22.5 %) were on dual anti-platelet therapy. Patients with carotid artery disease were significantly more likely to be on dual therapy (41 % versus 9 %, *p* < 0.001). 116 patients (45.3 %) were on anti-coagulation therapy. Of those on Warfarin (*n* = 100), the mean INR was 2.4 (1.2–3.7). 78 % had an INR between 2 and 3. 14 % had a sub-therapeutic INR and the remaining 8 % had a supra-therapeutic INR level. One quarter of patients on anti-coagulation (*n* = 28; 24 %) were also on anti-platelet therapy. Of patients with atrial fibrillation (*N* = 97), 83.5 % (81/97) were on anticoagulation at 6 months. The remaining patients (16/97; 16.5 %) were not on anticoagulation due to a contra-indication (12), patient too unwell (3) or patient refusal (1).

#### Lipid-lowering and anti-hypertensive medications

At follow-up, almost all patients (242; 94.5 %) were on lipid-lowering therapy and three-quarters of patients (*N* = 190; 74.2 %) were on anti-hypertensive therapy. Almost half of these patients were on single agent therapy, with a further one-third (33 %) on dual anti-hypertensive therapy. Remaining patients were on three (32; 16.8 %) or four (5; 2.5 %) agents. Those most commonly prescribed anti-hypertensives were beta blockers (*N* = 101, 39.5 %), angiotensin-converting-enzyme inhibitors (*N* = 95, 37.1 %) and calcium channel blockers (*N* = 55, 21.5 %). Although patients on lipid-lowering therapy were more likely to reach lipid targets, patients on anti-hypertensive therapy had poor therapeutic control of blood pressure at 6 months (Table 3).

**Table 3:** Therapeutic control of lipids and office blood pressure at follow-up

	Proportion reaching target (%)			
	All patients	On lipid-lowering medication	No lipid-lowering treatment	*p*-value
Lipid targets^a^ (mmol/l)				
TC < 5.0	(200) 86.2	(196) 89.5	(4) 30.8	*p* < 0.001
TC < 4.5	(174) 75.0	(171) 78.1	(3) 23.1	*p* < 0.001
LDL < 2.5	(170) 76.9	(167) 79.5	(3) 27.3	*p* < 0.001
LDL < 1.8	(97) 43.9	(96) 45.7	(1) 9.1	*p* < 0.05
	All patients	On anti-hypertensive medication	No anti-hypertensive medication	*p*-value
BP targets (mmHg)				
BP <140/90	(91) 36.6	(62) 33.2	(29) 46.8	*P* = 0.05
BP <135/85	(69) 27.7	(46) 24.6	(23) 37.1	*P* = 0.06
BP <130/80	(40) 16.1	(24) 12.8	(16) 25.8	*p* < 0.05

*TC* total cholesterol, *LDL* low-density lipoprotein, *BP* blood pressure

^a^European Guidelines on cardiovascular disease prevention (version 2012) [15]

## Discussion

This study reveals suboptimal control of many risk factors post IS despite the availability of evidence-based therapies and clear guidelines for secondary prevention. This cohort was at high-risk for recurrent stroke, with prevalent baseline risk factors, two-thirds over the age of 65 years, and one-quarter having previous stroke. These findings raise concern for health professionals and should prompt more intensive management of risk factors.

Studies have shown an association between TC, LDL and elevated TG with IS risk, especially among atherosclerotic and lacunar stroke subtypes [21]. Although mean lipid levels recorded at 6 months were not excessively high, many results were not at target. Only three-quarters of our cohort had TC at target (<4.5 mmol/L) with a similar proportion (77 %) having LDL at target (<2.5 mmol/L). However, these results compare favourably with those from EUROASPIRE III (49 and 55 % respectively at target) [8] and with results from the stroke-specific module of EUROASPIRE III [10], likely due to the higher uptake of lipid-lowering medications in our group. Outside the trial setting, there is currently no published data available on the adequacy of control of lipid levels specific to IS. Although the optimal LDL level for secondary stroke prevention is unclear, there is evidence from the SPARCL study that tight control (<1.8 mmol/L; achieved by only 44 % of our cohort) reduces recurrent IS risk by one quarter [22] and European guidelines now advise this target for high-risk patients [15].

Diabetes is independently related to a greater risk of IS (adjusted risk ratio, 2.26) [23]. For diabetic patients in ASPIRE-S with suboptimal control (HbA1c ≥ 7, 28 %), it is unknown (without access to patient notes at follow-up) whether the treating physician opted for less strict control in light of poorly tolerated hypoglycaemic episodes in some patients. Optimal target HbA1c levels in diabetic patients still remain the subject of much debate, and in most cases is individualised to particular patient characteristics [24]. Only 1.5 % of non-diabetic patients had FG ≥7 mmol/L. Four percent of patients had FG within the impaired fasting glucose range (≥6.1 mmol/L), but this rose to almost 10 % when the latest American Diabetes Association definition (≥5.6 mmol/L) was applied [16]. Diagnoses were not made on the basis of a single FG level in ASPIRE-S but it is known that early dietary and lifestyle modifications can delay or prevent the development of diabetes in patients with impaired FG [25].

The prevalence of obesity in the developed world has substantially increased over time [26]. Results from the Physician’s Health Study reported a significant increase in the relative risk of stroke with each unit increase of BMI, independent of the effects of other stroke risk factors [27]. Over two-thirds of our cohort had a BMI of ≥25 kg/m^2^ with one quarter being classified as obese. These levels are higher than those reported in the general Irish population (overweight 39 % and obese 18 %) [28] and likely contribute to other stroke/cardiovascular risk factors such as hypertension, hyperlipidaemia and diabetes. Two-thirds of participants had abdominal obesity. These results suggest that a more active role for sustained professional support and motivation in achieving successful weight reduction post stroke is needed, including (repeated) clear advice on weight loss from health professionals, which positively impacts on weight loss behaviour [29]. Although multiple successful weight reduction interventions are available, the ability of patients post stroke to engage in vigorous exercise programmes may be limited. Studies to explore the effectiveness of interventions such as group education or dietary changes for weight loss specifically after stroke are limited. The first randomized-controlled trial to evaluate the efficacy and safety of a weight management intervention in stroke survivors using the SystemCHANGE™ approach is currently underway [30].

The INTERSTROKE study reported a doubling in the risk for IS in current smokers (OR 2.09, 1.75–2.51) [3]. Although smoking rates are reportedly decreasing over time [2], 16 % were still smoking in our cohort at 6 months. This reflects a smoking cessation rate of 41 % (compared with 81 % in the Irish subgroup of EUROASPIRE III and 40 % in the stroke-specific module) [8, 10]. Although physician advice to stop smoking is the most important first step in the cessation process, this advice must be reiterated and reinforced by all health professionals [31]. Multiple pharmacotherapies for tobacco dependence have been shown to increase smoking cessation rates [32], and behavioural interventions can further increase the success of smoking cessation [33].

Although the mean BP reading was not excessively elevated, only 37 % of patients had BP ≤140/90 (similar to EUROASPIRE stroke module; 38 %). Such poor control of BP may reflect a lack of focus on secondary preventive initiatives for patients after stroke. The proportion at target was lower amongst patients with a previous diagnosis of hypertension and amongst those on anti-hypertensive therapy, reflecting the difficulty in treating hypertension in clinical practice. Recent European guidelines [5, 15] suggest a lower BP target of ≤130/80 in patients after IS, where tolerated. Only 16 % of our cohort achieved this target at 6 months and this proportion dropped to 13 % amongst those on anti-hypertensive therapy. Although these results reflect a single reading in patients, they are a reminder that better management of BP is required after IS including greater up-titration of medication dosages and use of multiple medication regimens in addition to lifestyle advice.

Ambulatory BP monitoring (reflecting multiple measurements) is known to provide a more accurate measure of BP control [34]. This is one of the first studies to describe ambulatory BP control post acute stroke and results clearly demonstrate greater overall BP control, compared with office readings. Better control of daytime (versus nightime) readings may reflect the timing of administration of antihypertensive medications (usually in the morning) which impacts on circadian control [35]. It may also reflect lack of attention placed by physicians on nightime BP control. Nightime BP control has been strongly linked to an increased risk of cardiovascular disease in multiple studies, including the Dublin outcome study of 5292 participants [36], which reported a relative hazard ratio (for cardiovascular mortality) of 1.21 for each 10-mm Hg increase in nightime systolic BP. This increased risk remained significant in older patients [36, 37].

Most IS patients are prescribed a combination of cardiovascular medications and this multi-factorial approach to secondary stroke prevention can result in a substantial reduction (of up to 80 %) in future stroke risk [38]. Although prescription rates of anti-thrombotic and lipid-lowering medications in our cohort were high, the prescription of anti-hypertensive medications appeared suboptimal. Despite positive findings from the pivotal PROGRESS study [39], a minority of patients in ASPIRE-S were on dual ACE I and diuretic therapy. However, a significant proportion of patients had hypertension (58 %), atrial fibrillation (40 %) and/or heart disease (30 %) at baseline and were likely already established on appropriate medications with anti-hypertensive effects (such as beta-blockers or CCBs) prior to their stroke.

This study has many strengths including its prospective design, large sample size, high rate of follow-up, in-hospital assessment of patients to verify eligibility for inclusion and a standardised follow-up assessment at 6 months modelled on previous robust EUROASPIRE surveys. There is little information in the stroke literature on the adequacy of secondary prevention after IS and much of the findings from ASPIRE-S are therefore novel. The recent EUROASPIRE III stroke-specific module (including four countries) reported some comparable findings but was retrospective, excluded IS patients over 80, had a broader follow-up period (6–36 months), used self-reported definitions of vascular risk factors and unhealthy lifestyle habits, did not include ambulatory BP monitoring and had limited generalizability.

### Limitations

The majority of the findings from ASPIRE-S are novel within the Irish stroke setting (and further afield), but there are some limitations to this study. Participants were recruited over 12 months as a representative (not consecutive) sample of ischaemic stroke patients in North Dublin. However, no attempt was made to be selective in any way and this cohort compares favourably in many ways with that recruited in 2006 as part of the North Dublin Population Stroke Study (NDPSS) [11]. Although the ASPIRE-S sample of three hospitals reflects the north Dublin population well, results may not be representative of the population nationally. It may be that the burden of cardiovascular risk factors is particularly high in north Dublin. Higher prevalence of cardiovascular risk factors has previously been linked with social disadvantage [40, 41] and the north Dublin population includes several communities where social disadvantage is common. Furthermore, although ASPIRE-S has a large overall sample size, the statistical significance of some results may have been limited by small numbers within sub-cohorts.

## Conclusion

Given the strong evidence from consensus of expert opinion for multi-dimensional risk factor management in secondary stroke prevention [38] it is imperative that tailored programmes of care (including risk factor control) be optimised. These should commence during the patients’ hospital admission with seamless transfer to the community. Over recent years there have been significant advances in the standardisation of clinical guidelines for stroke care, however the introduction of national stroke clinical guidelines alone is insufficient to improve health care quality [42]. For efficacy, guidelines should be incorporated into quality assurance cycles with education programmes and local feedback [43]. Results from ASPIRE-S promote awareness of the importance of ongoing surveillance of cardiovascular risk in patients after IS and prompt further roll-out of similar surveys internationally. This should encourage the establishment of local policies which support comprehensive, professional, multi-disciplinary secondary preventive initiatives, accessible to all survivors of stroke.
